# Early behavioral and developmental interventions in ADNP‐syndrome: A case report of SWI/SNF‐related neurodevelopmental syndrome

**DOI:** 10.1002/mgg3.1230

**Published:** 2020-04-10

**Authors:** Amelle Shillington, Ernest Pedapati, Robert Hopkin, Kristen Suhrie

**Affiliations:** ^1^ Cincinnati Children's Hospital Medical Center Cincinnati OH USA

**Keywords:** ADNP, autism spectrum disorder, congenital diaphragmatic hernia, risperidone, SWI/SNF

## Abstract

**Background:**

Autism spectrum disorder (ASD) affects approximately one in 59 children. Variants in the activity‐dependent neuroprotector homeobox *ADNP* (OMIM #611386) gene may be one of the most common single‐gene causes of syndromic ASD. Most patients diagnosed with ADNP syndrome have ASD as a comorbidity, and all patients have mild‐to‐severe intellectual disability.

**Methods/Case Report:**

We present a case report of a patient diagnosed with ADNP syndrome at 2.5 years of age. The patient has many of the key features of the syndrome, including ASD, global developmental delay, behavioral problems, congenital heart defect, early tooth eruption, and vision problems. The patient's initial presentation included congenital diaphragmatic hernia (CDH), which has not been previously reported in this condition.

**Results:**

The patient exhibited frequent behavioral outbursts and was initiated on antipsychotic medication with near‐complete resolution of symptoms allowing her to engage more fully in early intervention therapies leading to progress in language acquisition.

**Conclusion:**

This short report provides guidance for antipsychotic medication dosing to improve early intervention outcomes. This is the first report of CDH in this syndrome.

## Graphical abstract

ADNP is a regulator of the SWI/SNF chromatin remodelling complex.

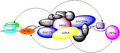

## 1 BACKGROUND

Autism Spectrum Disorder (ASD) is a developmental disorder characterized by impairments in social communication, along with restrictive interests and repetitive behaviors that are typically present by 2 years of age. ASD currently affects approximately one in 59 children, and the incidence has been on the rise (Wingate et al., 2014).

Both genetic and environmental factors are thought to be causative in the etiology of ASD which lead to changes in neuronal development, growth of the brain, and synaptic connectivity. As next‐generation sequencing (NGS) becomes more available and more affordable, many families along with their physicians are electing to pursue NGS testing to elucidate a specific molecular diagnosis for patients with ASD. It is reported that more than 2,000 genes are involved in ASD, and that no one gene is likely to explain more than 1% of cases (De Rubeis et al., 2014). The rarity of any one genetic mechanism of disease can make it very difficult to develop interventions and care plans for patients when there may be only a handful of patients reported to have the same genetic change.

**FIGURE 1:**
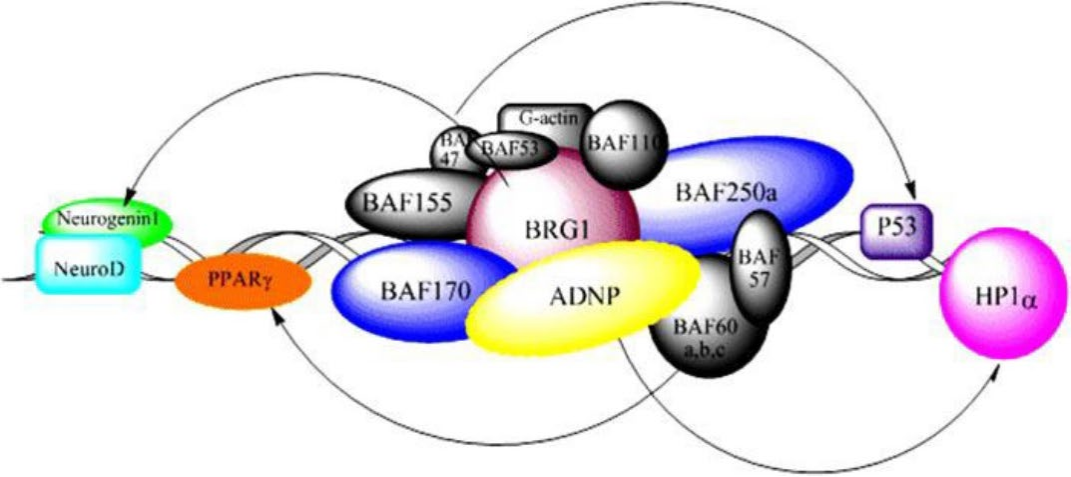
The SWI/SNF chromatin remodeling complex *Source*: Mandel et al (2007)

One gene that is commonly sequenced as part of NGS evaluation is the activity‐dependent neuroprotector homeobox (*ADNP*) gene, and may be one of the most common single‐gene causes of ASD; mutations in this gene are thought to explain the etiology of 0.17% of patients with ASD (Helsmoortel et al., 2014). The *ADNP* protein product is thought to be a regulator of axonal transport and dendritic spine plasticity at the synapse level (Gozes, Patterson, et al., 2017; Gozes, Patterson, et al., 2017), specifically functioning as a regulator of the essential chromatin‐remodeling complex, SWItch/sucrose non‐fermentable (SWI/SNF; Mandel & Gozes, 2007; Figure 1).

The connection between ADNP and the SWI/SNF complex is clinically relevant, as genetic disorders within this family of genes are well described and have neurodevelopmental feature overlap. The C‐terminal of ADNP directly interacts with ARID1A*,* SMARCA4, and SMARCC2, three essential components of the SWI/SNF complex. ADNP appears to be a regulator of SWI/SNF function, thus ultimately impacting chromatin remodeling which is essential for postmitotic activity‐dependent dendritic outgrowth and axonal development. Mutant ADNP protein appears to compete with wild‐type protein preventing optimal SWI/SNF complex function (Helsmoortel et al., 2014). Deficiencies in a working SWI/SNF complex lead to defects in neuronal development (Lessard et al., 2007).

Most patients diagnosed with ADNP Syndrome, also known as Helsmoortel–Van Der Aa Syndrome, have ASD as a comorbidity, and all patients have mild‐to‐severe intellectual disability (Arnett et al., 2018; Helsmoortel et al., 2014; Van Dijck et al., 2019). Other frequent findings include hypotonia, delayed developmental milestones including prominent speech delays of little to no words to some simple sentences, feeding problems in infancy followed by GI problems such as gastroesophageal reflux disease (GERD) and constipation as patients age, visual problems, and congenital heart defects (Helsmoortel et al., 2014). A unique feature of the syndrome is fully erupted dentition by the age of 1 year in over 80% of patients (Gozes, Patterson, et al., 2017; Gozes, Van Dijck, et al., 2017). Neuropsychiatric features appear to be common with reports of attention deficit hyperactivity disorder, anxiety disorder, and obsessive compulsive disorder (Helsmoortel et al., 2014). Dysmorphic features observed in patients include prominent forehead, high hairline, broad nasal bridge, thin upper lip, and smooth or long philtrum (Helsmoortel et al., 2014).

## 2 ETHICAL COMPLIANCE

Procedures followed were in accordance with the ethical standards of the responsible committee on human experimentation (institutional and national) and with the Helsinki Declaration of 1975, as revised in 2008. Protocol and procedures employed were reviewed and approved by the human subjects review committee. Informed consent from the family was obtained for publication of this clinical report.

## 3 CASE PRESENTATION

### 3.1 History of present illness (HPI)

This female patient was referred for Genetics evaluation at 18 months of age. She presented with speech delay, having no words at the time of initial evaluation. Additionally, she had gross motor delays and required braces for walking. She had poor weight gain. Her perinatal course was significant for prematurity, born at 36 weeks gestation. Additionally, she was born with congenital diaphragmatic hernia (CDH) that was not prenatally diagnosed but repaired shortly after birth. Postnatal echocardiogram demonstrated atrial septal defect. Ophthalmology exam diagnosed myopia and early pigmentary retinopathy.

### 3.2 Physical exam

**FIGURE 2:**
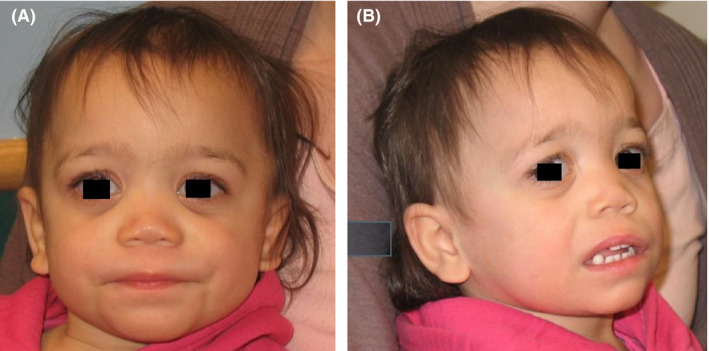
(a,b) Facial features of patient with ADNP syndrome, used with permission of family

Her physical exam was notable for thin sparse hair, downslanting palpebral fissures, marked epicanthal folds, posterior ear placement, anteverted nares, thin upper lip, smooth philtrum, and wide mouth accommodating full, early tooth eruption (Figure 2a,b). Additionally, she had a hemangioma on her right chest and an umbilical hernia. Her height, weight, and head circumference were all below the third percentile. Her developmental assessment showed inability to point to objects as well as difficulty communicating wants and needs, frequently resorting to screaming to get needs met. She did not play with toys in a typical fashion, but would bring toys to her mouth. She was noted to have frequent teeth grinding throughout the day. Parents reported ongoing problems with constipation.

### 3.3 Labs, imaging, and genetic testing

A brain magnetic resonance imaging (MRI) was completed and demonstrated nonspecific white matter changes consistent with hypomyelination or gliosis. A chromosomal microarray was ordered as part of her initial genetics workup, and was found to be normal. Definitive genetic testing was coordinated at 2.5 years of age, and clinical whole exome sequencing of the proband along with both parents was ordered to evaluate her constellation of symptoms. This was performed using capture with Agilent SureSelect ExomeXT and sequencing with an Illumina HiSeq 2500 on blood samples. Alignment to reference and variant calling were performed as described (Lee et al., 2014). Mean coverage was 20× of 95% of the target regions. This individual's exome DNA sequences were mapped and compared with human genome build UCSC hg19 reference sequence. Sequencing returned with a de novo pathogenic disease‐causing variant in the *ADNP* gene: *ADNP c*.2496_2499del (p.Asn832Lysfs*81). The c.2496_2499del (p. Asn832Lysfs*81) variant in the *ADNP* gene creates a frame shift starting at codon Asn832 in exon 4 of 4 exons. The new reading frame ends in a STOP codon at position 81. This variant was not found in dbSNP, gnomAD, and Exome Sequencing Project databases. This variant was reported in human gene mutation database (HGMD) (CD144180) as a disease‐causing mutation for Intellectual disability and/or autism and dysmorphic features and twice in ClinVar as a pathogenic variant. This variant has been previously reported in peer‐reviewed literature (Helsmoortel et al., 2014).

### 3.4 Developmental testing

With the understanding that most children with a diagnosis of ADNP syndrome present with ASD and intellectual disability, this patient was referred to Developmental Pediatrics for a developmental assessment which she completed at 3 years of age. She received an ADOS‐2 evaluation which showed moderate‐to‐severe concern for autism, and yielded an eventual diagnosis of ASD. She was administered the Mullen scales of early learning to assess her developmental abilities. This testing revealed specific deficits in visual reception, fine motor, and receptive and expressive language skills, meeting criteria for a diagnosis of global developmental delay.

### 3.5 Behavioral exam

At 3.5 years of age, the patient presented to psychiatry to evaluate maladaptive behaviors. The main concern expressed by parents was frequent, high pitched loud shrieks that occurred mainly when she was idle or when seeking attention. This shrieking often persisted for up to ten minutes continuously. Parents reported several concerns regarding the patient's mood. The patient would have episodes of intense fearfulness elicited by visual triggers. Triggers included certain individuals, colors of clothing, and certain toys or household items. Despite efforts of re‐exposure, the patient fears persisted upon presentation of the stimuli for several weeks. She also displayed periods of extreme moods including intense laughing spells and brief periods of irritability. Other than biting or pinching when upset seen commonly within this age group, the patient did not demonstrate any severe physical aggression or self‐injurious behaviors (i.e., head banging) associated with ASD. Her disposition was otherwise playful and curious and described as “happy”.

### 3.6 Early intervention

Due to her complicated neonatal course, including repair of CDH, this patient had the unique benefit of being followed by high‐risk pediatrics after discharge from the neonatal intensive care unit. As part of her care she began receiving early intervention services from 9 months of age due to her high‐risk status (and not so much due to her diagnosis of global development delay or ASD, diagnoses that were made at a later age). Indeed, she was evaluated by speech therapy before she had even developed words. At 18 months of age she began more intensive weekly speech therapy sessions, along with physical therapy and occupational therapy. At 2 years of age her speech assessment revealed that her skills were near that of a 10 months old; she was able to play a language/gesture game, produce three consonant sounds, and babble. She had at least two words (more, bye). She was not able to combine jargon and gestures, combine words and gestures, or jabber with inflection. At 3 years of age she began attending an ABA (Applied Behavioral Analysis) preschool three half days a week, and additionally had private speech and occupational therapy sessions. Private speech therapy enlisted the PROMPT technique (prompts for restructuring oral muscular phonetic targets), a therapy that engages a tactile‐kinesthetic approach using touch cues to a patient's jaw, tongue, and lips to manually guide them through language development (Rogers et al., 2006). At the time of this writing, the patient was just over 3 years of age and had mastered six words.

### 3.7 Pharmaceutical management

At 3 years of age the patient's behavioral symptoms led to increasing difficulty across several settings. The periods of fearfulness and mood changes, which were often unpredictable, would impede early intervention therapy sessions. The shrieking spells and sudden changes in mood could appear to bystanders as if the patient was in acute distress, and limited the ability of the patient to socialize with same‐age peers in public social settings. Symptoms endorsed on the 15‐item aberrant behavior checklist irritability (ABC‐I) subscale included “screams inappropriately”, “cries over minor annoyances”, “yells at inappropriate times”, “mood changes quickly”, “cries and screams inappropriately”, and “irritable and whiny”. After discussion of risks, benefits, and alternatives, the patient was started on a risperidone 0.1 ml (1 mg/ml solution) twice a day to target the shrieking spells and mood lability. Following 2 weeks of treatment with an atypical antipsychotic medication, our patient's family reported a partial improvement in the most troubling behaviors including inappropriate cries, unpredictable fearfulness, and mood lability. The patient's stool frequency decreased, which was noted as an adverse effect, and her polyethylene glycol dose was increased. The risperidone dose was increased to 0.2 ml twice daily with near‐complete resolution of symptoms without any further reports of adverse effects. At her 1 month follow‐up after initiation of medication to target behavior, the patient's symptoms had become markedly less intense and less frequent.

## 4 DISCUSSION AND CONCLUSIONS

Key features of ADNP syndrome are ASD and global developmental delay with most prominent deficits in speech and language. Evaluation of a large cohort of children with ADNP syndrome revealed that speech delay was present in 98.6% of individuals with a mean age of first words at 30 months of age with a range 7–72 months (Van Dijck et al., 2019). Indeed our patient's speech and language development seem to follow a similar course to the cohorts described in the published literature, however, her progress was slightly more advanced than the average. One key finding from the aforementioned cohort study is that a full 19% of patients had no language development at all. There does not appear to be a correlation in genotype to phenotype in the differences observed in language development in research cohorts (Van Dijck et al., 2019), thus one might hypothesize that early intervention is a key driver of improving developmental outcomes. Our patient had the unique opportunity to engage with speech therapy and other developmental therapies from 9 months of age, which may have provided a clear advantage to allow her to reach speech milestones slightly ahead of other ADNP‐syndrome peers.

Behavior problems are reported in 78% of children with ADNP syndrome (Van Dijck et al., 2019), including obsessive‐compulsive behavior, mood disorder, self‐injurious behavior, a high anxiety level, temper tantrums, and (verbally) aggressive behavior. Forty‐four percent of the individuals were reportedly hyperactive or easily distracted (Van Dijck et al., 2019). Several patients in the cohort were prescribed antipsychotic medications or stimulant medications to assist in the management of behavioral symptoms. Specific published pharmacological management guidelines have not been made available to date for ADNP syndrome.

Treatment with risperidone, a potent dopamine‐2, serotonin‐2A, and alpha‐1 receptor antagonist, has precedence in patients with ASD within in the family of SWI/SNF complex disorders. Risperidone is currently FDA approved for ages 5–16 years of age and is effective for the treatment of irritability in individuals with ASD (Janssen Pharmaceuticals IpF, 2012). The primary endpoint for the pivotal clinical trials that led to approval for use in ASD patients was a decrease in the ABC‐I subscale (Aman, Singh, Stewart, & Field, 1985). The ABC‐I was well matched to our patient's symptom presentation and subsequently demonstrated improvement following initiation of treatment. Pretreatment and routine follow‐up monitoring of extrapyramidal symptoms, metabolic laboratories, and anthropometrics is warranted and can warn of the development of long‐term complications. While our patient was less than 5 years of age at the initiation of therapy, the risk and benefits of using the medication in a patient less than 5 years of age were reviewed with the family, and ultimately a significant benefit was noted.

A unique feature of our patient's clinical presentation, which warranted consultation by the Genetics team prior to the findings of developmental delay, was CDH along with some unusual facial features and hypotonia. Congenital diaphragmatic hernia has not been described in cohorts of ADNP syndrome to date. Congenital diaphragmatic hernia, however, has been described in other ASD syndromes associated with chromatin remodeling genes in the SWI/SNF family. Sweeney et al. (2018) describe a patient diagnosed prenatally with left CDH, aortic arch hypoplasia, small left‐sided cardiac structures, and ventricular septal defect. She was described as having dysmorphic facial features and developmental delays. Molecular diagnosis confirmed a pathogenic *ARID1B* variant. Bartin et al. (2018) report a case of prenatally diagnosed hydrocephalus and macrocephaly, corpus callosum agenesis, a left diaphragmatic hernia, severe IUGR, and a ventricular septal defect. Sequencing analysis confirmed a pathogenic *ARID1A* mutation. Thus, we argue with this case report that CDH may indeed be a feature of ADNP syndrome given the reports of CDH in other mutations involving other genes in the SWI/SNF chromatin remodeling complex.

Despite the fact that features of ASD can be recognized as young as 12 months of age, and can be reliably diagnosed by age 3, the average child is not formally diagnosed with ASD until 5.5 years of age (Crane, Chester, Goddard, Henry, & Hill, 2016). By this age, gaps in learning, speech and language development have substantially increased between children with ASD and normally developing children. The opportunity for early, effective intervention is diminished the longer a diagnosis is delayed. Early intervention initiated before 12 months of age in our patient appears to have benefited the patient, and a specific genetic diagnosis at 2.5 years of age allowed more specific targeted interventions including PROMPT therapy and low‐dose antipsychotic medication to optimize continued participation in early intervention. Practitioners are encouraged to consider NGS sequencing in patients with global developmental delay, as a specific or early genetic diagnosis can impact targeted behavioral and developmental interventions.

## ETHICS APPROVAL AND CONSENT TO PARTICIPATE

Written consent to participate and written consent for photographs was received from the subject of this case report, this includes consent for publication.

## CONSENT FOR PUBLICATION

Written informed consent was obtained from the patient's parent for publication of this Case Report and any accompanying images and videos. A copy of the written consent is available for review by the Editor of this journal.

## CONFLICT OF INTEREST

None declared.

## AUTHORS' CONTRIBUTIONS

A.S. prepared the majority of the manuscript including the background, case report, and discussion. E.P. contributed manuscript content specifically discussing psychiatric pharmaceutical management. K.S. provided editorial review and was the original physician evaluating the patient. R.H. performed the genetic testing, and conferred the genetic diagnosis. All authors read and approved the final manuscript.

## Data Availability

Not applicable. All data generated or analyzed during this study are included in this published article.
